# Lockdowns and low- and middle-income countries: building a feasible, effective, and ethical COVID-19 response strategy

**DOI:** 10.1186/s12992-021-00662-y

**Published:** 2021-01-20

**Authors:** Oghenowede Eyawo, A. M. Viens, Uchechukwu Chidiebere Ugoji

**Affiliations:** 1 School of Global Health, York University, Victor Dahdaleh Building, Room 5022, 88 The Pond Road, Toronto, ON M3J 2S5 Canada; 2 Salem Clinic and Maternity, Salem City, Warri, Delta State Nigeria

**Keywords:** COVID-19, Lockdown, Low- and middle-income countries, Global health, Health policy, Health equity

## Abstract

Lockdowns can be an effective pandemic response strategy that can buy much needed time to slow disease transmission and adequately scale up preventative, diagnostic, and treatment capacities. However, the broad restrictive measures typically associated with lockdowns, though effective, also comes at a cost – imposing significant social and economic burdens on individuals and societies, especially for those in low- and middle-income countries (LMICs). Like most high-income countries (HICs), many LMICs initially adopted broad lockdown strategies for COVID-19 in the first wave of the pandemic. While many HICs experiencing subsequent waves have returned to employing lockdown strategies until they can receive the first shipments of COVID-19 vaccine, many LMICs will likely have to wait much longer to get comparable access for their own citizens. In leaving LMICs vulnerable to subsequent waves for a longer period of time without vaccines, there is a risk LMICs will be tempted to re-impose lockdown measures in the meantime. In response to the urgent need for more policy development around the contextual challenges involved in employing such measures, we propose some strategies LMICs could adopt for safe and responsible lockdown entrance/exit or to avoid re-imposing coercive restrictive lockdown measures altogether.

## Background

Time is what we want most, according to William Penn, but what we use worst. This has proven painfully true in the case of COVID-19, with time being the scarcest yet most squandered resource in our response efforts. While most countries around the world moved towards instituting regional or country-wide lockdowns after the effects of COVID-19 became undeniable, we have largely (besides a few notable exemplars e.g., Taiwan, New Zealand, Vietnam) [1–6], misused the time these lockdowns were supposed to bring us to adequately scale up prevention, diagnostic, research, and treatment capacity. As a result, many countries continue to endure an extended first wave or are now facing a second wave of infection. With an inability to sustain long-term lockdowns, given their severe economic, social, and health impacts [7–9], countries continue to struggle with formulating adequate strategies for deciding when and how to enter and exit lockdowns in a safe and responsible way.

This is particularly pressing in low- and middle-income countries (LMICs), who largely adopted the same lockdown response strategy as high-income countries (HICs) despite having inadequate access to basic resources (e.g., clean water, uninterrupted food supply chain, uninterrupted income (support), accessibility to refrigerators or power to store food, internet for online education/work) necessary to survive and comply with the restrictive measures associated with lockdowns. As a result, both the pandemic and these restrictive response measures have created massive burdens in LMICs that will provide little long-term benefits. Now faced with the prospect of having to wait much longer than HICs to receive their fair share of COVID-19 vaccine, LMICs may be tempted into thinking that returning to lockdowns provides the best response strategy in the meantime. We argue that LMICs should adopt strategies to prevent and control the spread of COVID-19 in ways that are more responsive to local contexts, provide more benefit for their populations, and prevent the need to re-enter lockdown. As Broadbent et al. have noted, ‘When we lockdown, we cause deaths in the developing world to prolong lives in the developed world… Lockdown is demonstrably not egalitarian in either its costs or its benefits… the costs will mostly fall, as ever, on the global poor’ [7]. In response, LMICs should prioritize their limited resources, infrastructure, and personnel on supporting and sustaining a package of structural and behaviour-based interventions that will better prevent the need to re-introduce coercive restrictive measures.

## Lockdown (what is it good for?)

First off, ‘lockdown’ – a term that is now widely used in the COVID-19 literature – is not a technical word in public health or law and currently lacks a universally accepted definition. Lockdown has been used within the literature to refer to measures associated with varying levels of restriction on movement, social interactions, and economic activities that are introduced in society, mostly by government, to reduce or completely eliminate disease transmission [10, 11]. COVID-19 related lockdowns as we have seen in LMICs or elsewhere for that matter involve region- or country-wide implementation of restrictive measures that exist along a spectrum of severity (from less intrusive to more intrusive) that is defined largely by geography/jurisdiction and, as a common thread, seeks to limit activities of, and association between, persons. Restrictive measures, such as quarantine, isolation, social distancing, curfews, school and business closures, and travel restrictions, are effective infection control and prevention measures, when used in a timely and comprehensive fashion, that minimize opportunities for person-to-person transmission and community spread of a virus [12]. These measures, however, are costly – imposing both significant individual and societal burdens [7–9]. Thus, in order to be epidemiologically and ethically justifiable, a lockdown needs to have a greater balance of benefits over burdens when being implemented, including considerations about the duration, breadth, and intensity of the restrictive measures involved.

In most societies, the employment of a lockdown strategy, as we have seen with COVID-19, is meant to be a first-line, time-limited pandemic response. Lockdowns can be an effective pandemic response strategy that allows us to buy time – and this is how they were sold to the public with the rallying cry to endure short-term pain in order to “flatten the curve” or “crush the curve” – but buying time for what? In HICs, the rationale for adopting a lockdown strategy was clear: it bought time to scale up response capabilities. Slowing down the transmission of the virus and maintaining surge capacity within the healthcare system bought them time by seeking to minimize infections and their consequences, while at the same time providing the opportunity to build up preventative (e.g., personal protective equipment for healthcare workers; masks for the general public; vaccine development), diagnostic (e.g., validating and producing testing kits; training contact tracing personnel or developing a tracing app), and therapeutic (e.g., drugs, ventilators) supplies.

There were many LMICs that rapidly moved towards implementing broad restrictive measures in early 2020. However, without due contextual consideration of their associated challenges and impacts, such efforts only seemed to delay the long-term burdens to come [13]. While the use of lockdowns did slow down viral transmission to some extent, the overall rationale for their use in LMICs is less clear especially in terms of the balance of the burdens over the benefits – unlike HICs who generally tend to have relatively robust healthcare system capacity, income support to allow people to make the choice to stay home, employment opportunities that allow a large number of people to work from home, and a telecommunications infrastructure that facilitates online education. Without the necessary capacity, infrastructure, and resources to maintain sustained compliance with a lockdown strategy, lockdown measures in LMICs risk leading to tragic consequences, e.g., starvation, economic ruin, neglect of other pressing health issues [14–16]. There is an urgent need for more research and policy development around how LMICs can develop lockdown entrance and exit strategies that are not only feasible, effective, and ethical [17, 18], but also would prevent the need to go back into lockdown.

## LMIC lockdown strategies: entrance, exit, and prevention

The robust implementation of the restrictive measures that compose a lockdown strategy must consider both how we can impose *and* subsequently relax restrictive measures in a way that is *feasible* (i.e., easily or conveniently executed given contextual features), *effective* (i.e., successful in achieving the desired outcome), and *ethical* (i.e., seeks to limit the disproportionate burdens borne by the least advantaged). The key challenge for policy makers is achieving a lockdown strategy that can meet all three objectives, with the acknowledgement that successful pandemic response strategy requires more than a one-size-fits-all approach [19]. For instance, China’s authoritarian approach to COVID-19 response can be viewed as effective, but will not be feasible in other settings with different political and legal cultures. We propose some strategies that LMICs could employ in combination to ensure long-term structures and approaches are in place to avoid re-imposing coercive lockdown measures or, if some form of lockdown is re-imposed, provide a way to conscientiously exit these restrictions. Some of these recommendations are arguably equally applicable to HICs that are struggling to mount a successful response, but they will be critical for LMICs given that they may have to deal with COVID-19 crisis for longer than HICs, who will most certainly receive vaccines earlier once these become available and have a better distribution infrastructure.

### Follow the science: the basics of infectious disease epidemiology and infection prevention and control remain true and should be at the centre of pandemic response

We have repeatedly seen within both HICs and LMICs the absolute centrality of governance and political decision-making as a determinant of pandemic response success. Those politicians who astutely and carefully followed the science in a prompt fashion were more likely to have less infection, death, and economic destabilization [3, 4, 20, 21]. We know that to effectively slow and eliminate disease transmission, the connecting links in the chain of infection have to be broken. This principle of infectious disease control has always been and remains true even for COVID-19 [22]. The unprecedented nature and novelty of the SARS-CoV-2 virus should not make governments ignore science and the basics of infectious disease control and prevention. Instead, this principle in concert with the local epidemiological data and other contextual considerations should be used to inform response measures, including advice on testing, quarantine, contact tracing, physical distancing, hand hygiene, use of masks, and how to open up schools and businesses safely.

### Promote prevention by focusing on strategies of adherence rather than compliance

In most LMICs, the implementation of restrictive measures in lockdown largely adopted a *compliance approach* that sought to produce passive behaviours through prohibitions and punishments – where the response interventions focused on negative behaviours, constraining choices, and coercive enforcement. In relaxing restrictive measures, LMICs should instead adopt an *adherence approach* that continues to reduce people coming into close contact, but instead focuses on encouraging enabling and supportive behaviour, where the selection of response interventions are positive, proactive, and enhancing of the skills or knowledge that can help people mitigate risk or reduce harm [23]. Central to this will be health promotion and protection education about COVID-19, including advice on how people can protect themselves and others (e.g., disseminated through TV, radio, social media, and cellphone text messaging alerts) and small-scale government-led infrastructure development projects to improve access to clean water that would enable people to follow basic infection prevention and control practices, e.g., encourage adherence to preventative measures, such as regular hand washing, which due to lack of access to clean water is currently difficult for many to practice in most LMICs. Instead of focusing on superficial and short-term interventions that neglect contextual realities and breed antipathy, LMIC governments need to invest in long-term interventions that command wide support and adherence, such as dependable and uninterrupted access to clean water that builds up public health infrastructure – which also contributes to addressing other pressing health problems (e.g., water and food-borne diseases) in these settings.

### Community engagement and trust building are central

The speed – often before a COVID-19 case was detected – in which some LMICs implemented lockdown measures (e.g., curfews, travel bans, school and business closures) did not allow for sufficient community engagement around the severity, breadth, or duration of restrictive measures. Moreover, the deficit of trust that exists between citizens and government as a result of political corruption and inadequate investment in the health system means that the imposition of any response measures, especially coercive measures, would be looked on with suspicion and derision. There is now an important opportunity for government officials to focus on community engagement and building trust through seeking to understand their unique contextual factors and using that information to tailor local strategies that would enhance community buy-in and acceptance of the strategies by focusing on interventions that prevent infection and the likelihood of needing to return to lockdown [24]. Increased engagement with communities can also create an opportunity to address issues of misinformation that is fuelling stigma and discrimination against people infected with the SARS-CoV-2 virus, including impeding testing and care-seeking behaviour [25–27].

### LMICs should take a targeted approach to testing

Accurate and rapid diagnostic test results for SARS-CoV-2 provide a key means to evaluate the effectiveness of pandemic response, as well as using the information to develop a more targeted approach to prevent further spread of COVID-19 through contact tracing. Nevertheless, significant technical limitations still exist across all contexts, including concerns regarding the diagnostic accuracy of available testing options [28, 29], and, in LMICs, large-scale testing will not be feasible given limited infrastructure, diagnostic supplies, and technical capacity. Instead, LMICs should focus on free, point-of-care targeted testing – demand-driven by jurisdictional epidemiological reports/statistics – among symptomatic individuals.

### Recruit and train more healthcare and support workers in the short term

Until there is widespread vaccination, the incidence of COVID-19 cases could rise dramatically and overwhelm the capacity of the healthcare system, especially when healthcare worker capacity has not been sufficiently strengthened to manage the disease in the long-term. LMICs should focus on practical, short-term solutions, including (i) fast-track medical/nursing students to start now in the field and then return to finish up their degrees later; (ii) organize short infection control courses for other allied health professionals to provide support on small non-technical tasks; and (iii) task-shift and train local community members who can be gainfully engaged to carry out contact tracing activities with some oversight. This latter strategy has been successfully applied in LMICs in the area of HIV prevention, treatment, care, and support [30].

## Conclusion

Lockdowns strategies – including choices around when and how to enter and exit lockdowns – are, fundamentally, political decisions. COVID-19 has significantly highlighted the importance of the political determinants of health [31], and the role of political leadership and governance in the selection and implementation of pandemic response measures that are feasible, effective, and ethical. For LMICs, how the time gained from the adoption of lockdown’s restrictive measures in broad or relaxed forms is utilized will ultimately determine how safely we can get back to our normal lives, whatever the new ‘normal’ will be. Given that there is no one magic bullet to address COVID-19, political decisions should target a combination of science-based and context-informed strategies that are the foundations of a strong public health system to ensure LMICs can successfully prevent the need to go back into lockdown and contributes to the overall success of the local and global response to the pandemic.

## Data Availability

Not applicable.

## Abbreviations

HICs: High-income countries
LMICs: Low- and middle-income countries
